# Glucose transport as a design constraint in white-zone meniscus tissue engineering: a reaction-diffusion framework

**DOI:** 10.3389/fbioe.2026.1880978

**Published:** 2026-10-15

**Authors:** Tiya Patel

**Affiliations:** 1 Orthopaedic Research Laboratory, Rutgers - Robert Wood Johnson Medical School, New Brunswick, NJ, United States; 2 Department of Biomedical Engineering, Rutgers University - New Brunswick, New Brunswick, NJ, United States

**Keywords:** glucose transport, meniscus, model-derived diffusivity estimate, reaction-diffusion model, release kinetics, scaffold-assisted repair, tissue engineering - meniscus - meniscus replacement - biomaterial - cell, white-zone tear

## Abstract

The adult knee meniscus has a strong vascular gradient: the peripheral red zone is vascularized, whereas the inner white zone is largely avascular and has limited intrinsic healing potential after injury. Although biologic augmentation and scaffold-based strategies are being explored to improve meniscal repair, it remains unclear whether modifying local glucose transport can improve glucose availability at deep white-zone tears. This study uses a one-dimensional reaction-diffusion framework to model normalized glucose concentration from a vascularized red-zone boundary toward a simulated 2.5 mm white-zone tear. Glucose diffusivity values were selected from reported meniscus glucose-diffusion measurements under different compressive strain conditions, and the model compares native diffusivity cases with an augmented sustained-source case representing a hypothetical scaffold- or microchannel-assisted transport scenario. The framework reports threshold-free glucose profiles, mathematical fractional-restoration comparison levels (C/C0 = 0.20, 0.30, 0.40, and 0.50), source-release kinetics, tear-depth sensitivity, initial-condition sensitivity, extended simulation windows, and model-derived diffusivity enhancement estimates. The comparison levels are not validated healing thresholds or clinical targets. Native glucose diffusivity cases remained below C/C0 = 0.30 at a tear located 2.5 mm from the red-zone boundary, even when selected simulations were extended to 24 h. In contrast, an augmented sustained-source example reached C/C0 = 0.20, 0.30, and 0.40 within the 4-h transport window. Initial condition sensitivity showed that the main interpretation remained unchanged when the model was initialized with a reference steady glucose profile rather than a depleted domain. These results do not demonstrate biological healing, but they provide a hypothesis-generating framework for defining glucose transport experiments and planning future *ex vivo* validation studies.

## Introduction

The knee meniscus is a fibrocartilaginous structure that helps stabilize the joint, distribute load, absorb shock, and protect articular cartilage during daily movement and athletic activity (Makris et al., 2011; Mameri et al., 2022). Each knee contains a medial and lateral meniscus composed primarily of type I collagen, water, proteoglycans, and regionally organized extracellular matrix. The circumferential collagen arrangement helps the tissue resist hoop stress during compressive loading, but this mechanical specialization is paired with a biologically limited repair environment (Fox et al., 2012; Makris et al., 2011).

Only the peripheral portion of the adult meniscus is directly vascularized, while the inner white zone relies heavily on diffusion for oxygen, glucose, and soluble signaling molecules (Arnoczky and Warren, 1982; Fox et al., 2012; Travascio and Jackson, 2017). This vascular gradient is clinically important because tears closer to the red zone generally have greater repair potential than deep white-zone tears, where nutrient supply, cellular response, and vascular signaling are more limited (Mordecai et al., 2014; Vaquero and Forriol, 2016).

Meniscal injuries are common and clinically important because poorly healed tears alter joint mechanics and can accelerate cartilage degeneration. Although partial meniscectomy can reduce symptoms, preservation of native meniscal tissue is preferred when possible because the meniscus is important for long-term joint health and joint-load distribution (Makris et al., 2011; Mameri et al., 2022; Mordecai et al., 2014). For deep white-zone tears, repair often fails not simply because of surgical technique, but because the local microenvironment has limited access to the nutrients, cells, and biochemical cues needed for repair.

Recent work has renewed interest in biologic and scaffold-based augmentation strategies for meniscus repair. Growth factors, platelet-derived biologics, cell-based approaches, hydrogels, and porous or channel-based scaffolds have all been proposed to improve the healing environment, but clinical translation remains inconsistent (Petersen et al., 2005; Yan et al., 2021). At the same time, transport-focused studies show that meniscal diffusion and permeability depend on solute size, tissue structure, loading, depth, tissue layer, anisotropy, and regional composition (Kleinhans et al., 2015; Morejon et al., 2021; Schwartz et al., 2022; Travascio et al., 2020). These findings suggest that white-zone repair may be limited by a transport bottleneck that could potentially be modified through scaffold architecture, microchannels, or local supplementation.

Computational models are useful for isolating transport effects because diffusion, consumption, and local supplementation interact in ways that are difficult to separate experimentally. Travascio and Jackson (2017) previously modeled human meniscus nutrition and showed that vascular recession can alter glucose availability within the tissue. The present study builds on this transport-centered view but asks a more scaffold-oriented design question: how much must effective glucose diffusivity and/or local glucose-equivalent supplementation increase to alter normalized glucose concentration at a white-zone tear depth? The goal is not to prove biological healing directly, but to create a quantitative framework that generates experimentally testable transport predictions for scaffold design and *ex vivo* validation.

## Methods

### Model overview

A one-dimensional reaction-diffusion model was developed to simulate early glucose transport across the radial thickness of the meniscus. The domain extended from the peripheral red-zone boundary at x = 0 mm to the inner white-zone margin at x = 3 mm. A tear was placed at x = 2.5 mm to represent a deep white-zone injury. The state variable C(x,t) represents normalized glucose concentration, where C0 = 1 is the imposed glucose concentration at the vascular red-zone boundary. The previous vascular-response variable was removed from the revised framework so that the analysis focuses on the transport process that can be assigned a specific molecule, diffusivity, and consumption term. Angiogenic and anti-angiogenic biology are therefore discussed only as future biological processes that are not simulated in the present glucose transport model.

### Physiological interpretation of normalized glucose comparison levels

This study uses C/C0 to describe glucose availability relative to the vascular red-zone boundary. In this normalized framework, C/C0 = 1 represents the glucose level imposed at the vascular boundary, while lower values represent fractional glucose restoration deeper in the meniscus. This normalization is useful because the absolute glucose concentration at the tissue boundary can vary with culture media, synovial fluid composition, vascular supply, and experimental setup. Therefore, C/C0 should be interpreted as relative glucose availability, not as a direct clinical measurement of healing.

The values C/C0 = 0.20, 0.30, 0.40, and 0.50 shown in Table 1 are mathematical comparison levels rather than validated biological thresholds. The exact glucose concentration that guarantees white-zone meniscal healing has not been established, and repair is affected by oxygen tension, glucose availability, inflammatory signaling, cell phenotype, matrix synthesis, mechanical loading, and vascular access. These values are retained only to compare fractional glucose restoration across model conditions. They should not be described as healing thresholds, clinically meaningful targets, or scaffold requirements. Future explant experiments should replace these normalized comparison levels with measured biological endpoints such as cell viability, matrix synthesis, inflammatory-response markers, or oxygen/glucose thresholds (Adesida et al., 2006; Adesida et al., 2007; Hennerbichler et al., 2007; Szojka et al., 2019; Travascio and Jackson, 2017).

### Governing equation

Glucose transport was represented using a reaction-diffusion equation with effective glucose diffusivity, first-order glucose consumption, and a localized scaffold source. Reaction-diffusion models are commonly used to represent transport with local loss or production terms in biological tissues, and meniscus nutrition models have used transport-based formulations to examine how vascular supply and diffusion shape glucose availability within the tissue (Travascio and Jackson, 2017; Travascio et al., 2020). The glucose equation was written

$$\text{dC}/\text{dt}=D\_\text{glu }d^{2}C/{\text{dx}}^{2}-k\_\text{glu }C+S\_\text{glu}\left(x,t\right)$$ (1)

In Equation 1, D_glu is the effective glucose diffusivity being tested, k_glu is the first-order glucose consumption rate, and S_glu(x,t) is a localized glucose-equivalent source centered at the tear. This formulation intentionally uses one specified nutrient species. If the framework is later applied to oxygen, glutamine, cytokines, or growth factors, D_glu and k_glu must be replaced with molecule-specific diffusivity and consumption or reaction parameters.

### Scaffold source term and release kinetics

The local source term was included to represent a biomaterial-assisted supply of glucose-equivalent soluble support near the tear. Earlier versions treated S0 as a constant non-depleting source. That assumption is computationally convenient, but real scaffold and hydrogel delivery systems often show burst release, diffusion-controlled release, swelling effects, or degradation-dependent depletion rather than indefinite constant delivery (Higuchi, 1961; Huang and Brazel, 2001; Siepmann and Siepmann, 2012; Whitaker et al., 2001).

$$S\_\text{glu}\left(x,t\right)=S0\;I\left(\left|x-x\_\text{tear}\right|\leq w/2\right)\;g\left(t\right)$$ (2)

In Equation 2 above, w is the source width and g(t) describes release kinetics. Three release cases were compared: a sustained source, g(t) = 1; a first-order depletion source, g(t) = exp(-t/tau); and a burst-decay source, g(t) = f exp(-t/tau_fast) + (1 - f) exp(-t/tau_slow). The sustained source should be interpreted as an optimistic upper-bound design case. The time-dependent depletion cases test whether predicted glucose restoration changes when the scaffold source declines over time.

### Boundary and initial conditions

The primary boundary condition was C(0,t) = C0 = 1 at the red-zone side of the domain. This boundary approximates glucose supply from the vascularized peripheral meniscus, where blood vessels enter the outer tissue (Arnoczky and Warren, 1982; Travascio and Jackson, 2017). At x = 3 mm, a zero-flux radial boundary, dC/dx = 0, was imposed. This inner boundary does not mean that synovial fluid is biologically absent. Instead, it reflects the geometry of the simplified one-dimensional radial model. The model coordinate runs from the vascular red-zone boundary toward a deep white-zone location and does not include the superior or inferior articular surfaces that contact synovial fluid. Explicit synovial-fluid supply would require a two- or three-dimensional model with surface boundary conditions or a separate convection-diffusion term. Excluding synovial supply from the main one-dimensional radial model makes the estimate conservative and keeps the analysis focused on red-zone-to-white-zone radial transport. Future models should add synovial surface exchange once experimental surface glucose boundary values are available (Travascio and Jackson, 2017).

The main simulations used C(x,0) = 0 throughout the interior as a depleted starting condition for comparing early transport rescue. Because this is not a fully physiological baseline, an additional initial-condition sensitivity analysis was performed using the analytical no-source steady glucose profile for the reference diffusivity as the starting condition. This second initialization represents a more physiological pre-existing glucose gradient before augmentation. The same no-flux radial inner boundary was used in both initial-condition cases.

### Numerical implementation and verification

The domain was discretized into 151 spatial nodes with dx = 2.0 × 10^−5^ m. The model was solved using an implicit finite-difference scheme, which was selected to avoid numerical instability during parameter sweeps, release-kinetics comparisons, initial-condition sensitivity testing, and extended time-window simulations. The nutrient update was solved in matrix form at each time step as:

$$C^{n+1}={\left[I-\Delta t\left(D\_\text{glu}\;L-k\_\text{glu}\;I\right)\right]}^{-1}\;\left[C^{n}+\Delta t\left(D\_\text{glu bC}+S\_\text{glu}\left(t\right)\right)\right]$$ (3)

In Equation 3, L is the finite-difference Laplacian matrix, bC contains the fixed red-zone boundary contribution, and S_glu(t) is the source vector at the current time step. A time step of Δt = 60 s was used for the revised analyses.

Numerical verification was performed by comparing the computational solution to the analytical steady-state solution for the no-source reaction-diffusion case with fixed red-zone boundary and zero-flux inner boundary. For D d^2^C/dx^2^ - kC = 0, C(0) = C0, and dC/dx(L) = 0, the analytical solution is:

$$C\left(x\right)=C0\;\cosh\left(\lambda \left(L-x\right)\right)/\cosh\left(\lambda L\right),\lambda =\sqrt{\left(k/D\right)}$$ (4)

This verification step tests done using Equation 4, tests whether the numerical solver reproduces a known reaction-diffusion solution under simplified conditions. The verification does not biologically validate the model, but it provides evidence that the numerical implementation solves the intended equations correctly. This distinction between numerical verification and biological validation is important for computational biomechanics credibility (Anderson et al., 2007). Convergence was also evaluated using the relative change between successive glucose profiles using Equation 5 shown below:

$$\text{epsilon}\_C={\left|\left|C\left(t\_n\right)-C\left(t\_\left\{n-1\right\}\right)\right|\right|}_{2}/{\left|\left|C\left(t\_n\right)\right|\right|}_{2}$$ (5)

### Model parameters and rationale

Table 2 summarizes the model parameters. The most important change in this revision is that the transport variable is now specified as glucose. The three native diffusivity values were selected from the glucose diffusion measurements of Kleinhans et al. (2015), who measured apparent glucose diffusion in porcine meniscus fibrocartilage using a one-dimensional steady-state diffusion chamber. The selected values correspond to axial-central specimens at 20%, 10%, and 0% static compression, respectively. This gives a consistent molecule, region, and experimental framework for D_low, D_ref, and D_upper. Human meniscus fluorescein measurements by Travascio et al. (2020) are cited only as supporting context because fluorescein is a small solute of comparable size, but the model diffusivity values used here are taken from the glucose-specific study.

**TABLE 1:** Fractional glucose-restoration comparison levels used in the model.

Normalized comparison level	Interpretation	Purpose in this model
0.20	Low partial glucose restoration	Detects whether a deep tear receives any increase above poor native transport
0.30	Intermediate mathematical comparison level	Used as the main comparison point while explicitly not being treated as a biological or clinical healing threshold
0.40	Moderate fractional restoration	Tests whether an augmented condition produces stronger transport improvement beyond the minimum comparison value
0.50	High fractional restoration	Serves as a more demanding mathematical comparison level approaching one-half of the red-zone boundary value

**TABLE 2:** Model parameters, values, sources, experimental basis, and rationale.

Parameter	Value	Units	Original source/experimental basis	Model role/rationale
L	3 × 10^−3^	m	Model domain selected to represent radial distance from vascular boundary to inner white-zone margin	Radial meniscus transport length
D_low	0.79 × 10^−10^	m^2^/s	Kleinhans et al. (2015): glucose, porcine meniscus, axial-central specimens, 20% static compression, 1D steady-state diffusion chamber	Lower glucose diffusivity case under compressed conditions
D_ref	1.10 × 10^−10^	m^2^/s	Kleinhans et al. (2015): glucose, porcine meniscus, axial-central specimens, 10% static compression, 1D steady-state diffusion chamber	Reference glucose diffusivity case used for fold-change comparisons
D_upper	1.68 × 10^−10^	m^2^/s	Kleinhans et al. (2015): glucose, porcine meniscus, axial-central specimens, 0% compression, 1D steady-state diffusion chamber	Upper native glucose diffusivity case under uncompressed conditions
D_aug	4D_ref	m^2^/s	Not a literature measurement; model-derived augmented transport case	Exploratory scaffold/microchannel transport enhancement example, not a clinically applicable requirement
k_ref	1 × 10^−4^	s^−1^	Phenomenological first-order glucose consumption value; varied in D-k analysis	Gives 1/k_ref = 2.78 h, half-life = 1.93 h, and √(D_ref/k_ref) = 1.05 mm
C0	1.0	Normalized	Fixed boundary concentration normalized to red-zone glucose supply	Sets C/C0 = 1 at the red-zone boundary
x_tear	2.5 × 10^−3^	m	Model-selected deep white-zone tear distance	Primary tear location
S0	2 × 10^−5^	C0/s	Model-derived local glucose-equivalent supplementation value; compared across release kinetics	S0/k_ref = 0.20 C0 and source-only C(4 h) = 0.153 C0, so the source alone cannot force crossing of the 0.30 level
Source width	0.5 × 10^−3^	m	Model-selected tear-centered source window	Localized supplementation region
tau	2	h	Model release parameter guided by first-order depletion behavior in biomaterial delivery literature	Time constant for first-order source depletion
tau_fast, tau_slow	0.5, 8	h	Model release parameters representing burst and sustained phases	Fast and slow time constants for burst-decay release

The augmented diffusivity D_aug = 4D_ref is not a literature-measured material property and should not be interpreted as a clinically applicable scaffold requirement. It is a model-derived exploratory case used to test how much increasing effective glucose transport could alter tear-site concentration. Similarly, the first-order consumption rate k_ref and source strength S0 are phenomenological values used for model exploration. Their quantitative scales are reported to improve transparency, and the D-k design-space analysis varies consumption so the conclusions do not depend on one assumed k value.

### Simulation conditions and outcome measures

Four primary transport cases were simulated: lower glucose diffusivity, reference glucose diffusivity, upper glucose diffusivity, and an augmented sustained-source design example. The augmented example combined a four-fold increase over D_ref with localized supplementation. Primary outcomes included final C/C0 at the tear site, threshold-free glucose profiles, crossing times for the mathematical comparison levels C/C0 = 0.20, 0.30, 0.40, and 0.50, the model-derived D/D_ref estimate required to reach each comparison level, sensitivity to tear depth, sensitivity to release kinetics, and sensitivity to the initial condition. To address whether the 4-h window shaped the interpretation, selected cases were also simulated to 24 h. A two-parameter design-space analysis varied D/D_ref and k/k_ref to determine whether the transport estimate changed when glucose consumption differed from the reference value.

## Results

The glucose diffusivity cases produced low glucose availability at a deep tear site after 4 h (Figure 1). At x = 2.5 mm, the lower glucose diffusivity case produced C/C0 = 0.044, the reference case produced C/C0 = 0.084, and the upper glucose diffusivity case produced C/C0 = 0.163. None of these native glucose transport cases reached C/C0 = 0.20 or C/C0 = 0.30 within the 4-h transport window. In contrast, the augmented sustained-source design example produced C/C0 = 0.468 at the tear site after 4 h.

**FIGURE 1:**
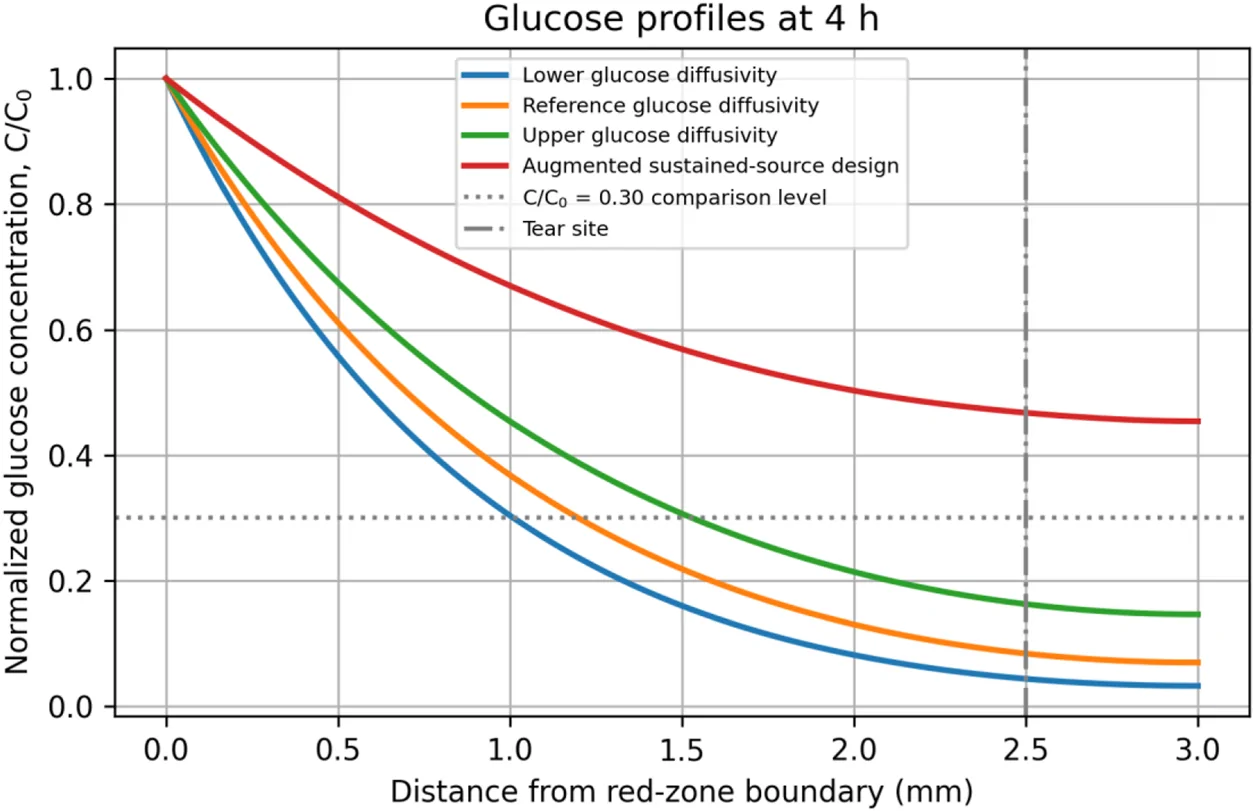
Glucose concentration profiles after 4 h. Native glucose diffusivity cases remain below the main C/C0 = 0.30 comparison level at the 2.5 mm tear, while the augmented sustained-source design example produces substantially higher glucose availability. Full concentration profiles are reported so the reader can compare transport behavior without relying only on one cutoff.

The comparison-level sweep showed that the augmented sustained-source design example reached C/C0 = 0.20 at 65 min, C/C0 = 0.30 at 97 min, and C/C0 = 0.40 at 151 min (Figure 2). It did not reach C/C0 = 0.50 within 4 h. This pattern shows that the predicted transport improvement is not dependent only on the 0.30 comparison level. The augmented condition improves glucose availability across a range of fractional restoration values, while the native reference case remains below even the lowest comparison level.

**FIGURE 2:**
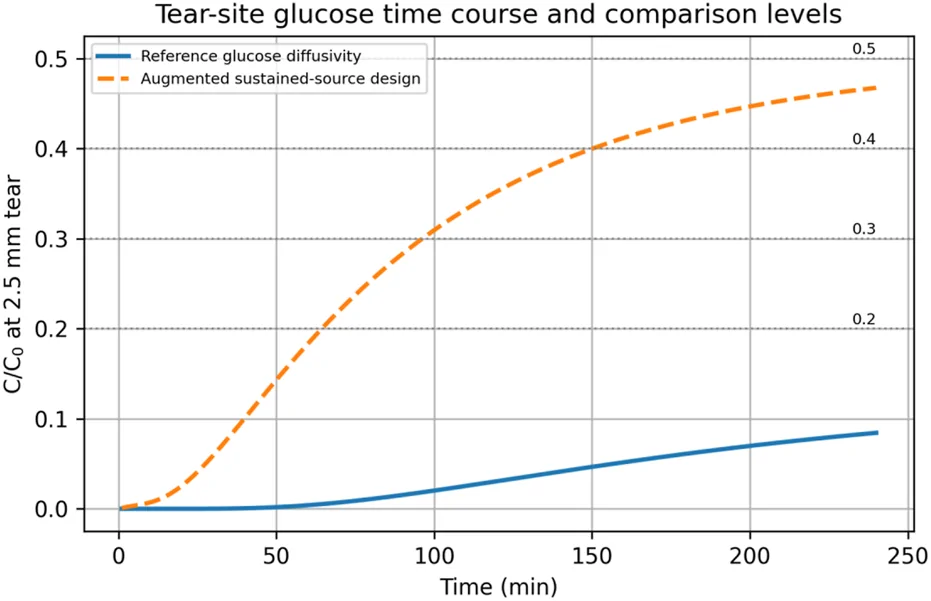
Tear-site glucose concentration over time with multiple fractional comparison levels. C/C0 = 0.30 is retained as an intermediate mathematical comparison point, but the model also reports C/C0 = 0.20, 0.40, and 0.50 so the interpretation does not rely on one arbitrary value.

The model-derived diffusivity analysis converted the model output into an estimate of the transport enhancement required under the stated assumptions (Figure 3). With no local source, reaching C/C0 = 0.30 at a 2.5 mm tear within 4 h required approximately D/D_ref = 2.63. With S0 = 2 × 10^−5^ C0/s, the required enhancement decreased to approximately D/D_ref = 2.33. More demanding comparison levels required larger transport improvements: reaching C/C0 = 0.40 required approximately D/D_ref = 3.23 with the same local source, and reaching C/C0 = 0.50 required approximately D/D_ref = 4.56. These values are model-derived estimates based on unvalidated assumptions, not clinically applicable scaffold requirements.

**FIGURE 3:**
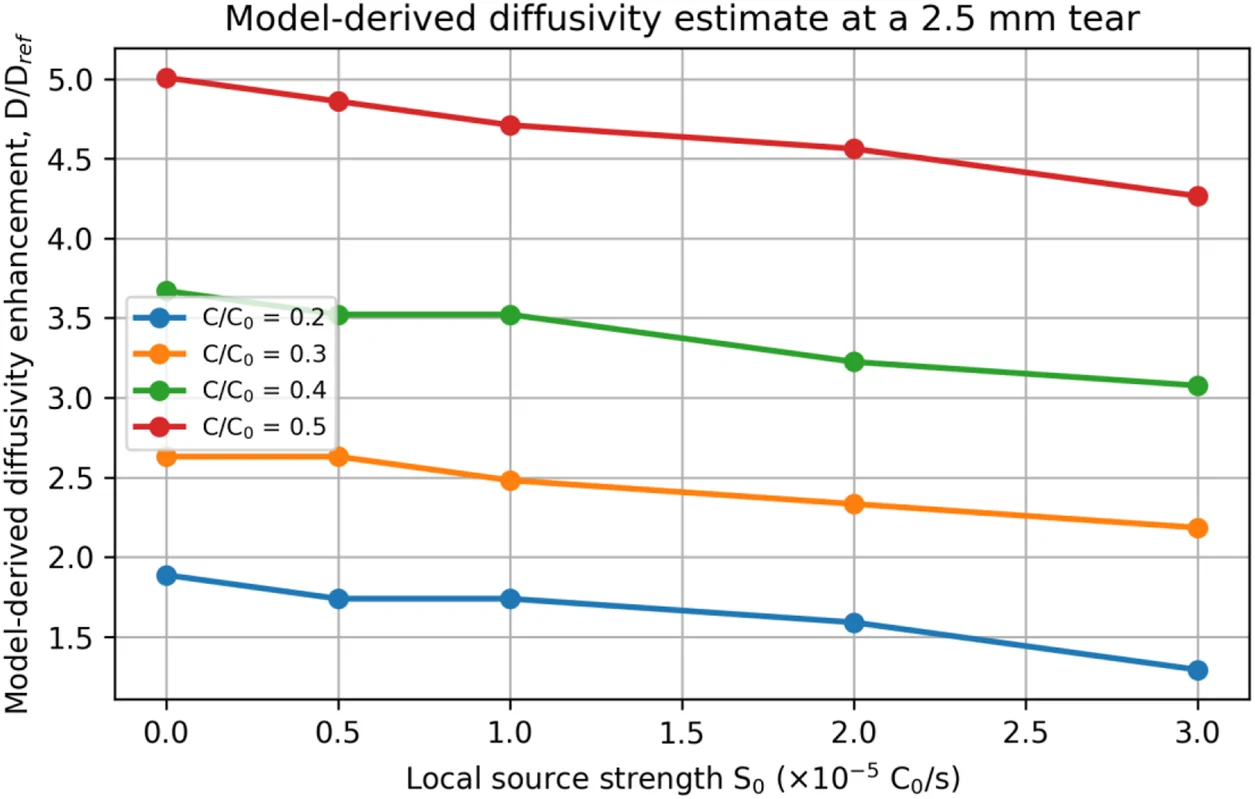
Model-derived diffusivity enhancement estimates for reaching fractional glucose comparison levels at a 2.5 mm tear after 4 h. Increasing local supplementation lowers the estimated diffusivity enhancement, but higher fractional glucose levels still require stronger transport improvement. These are model estimates, not validated scaffold design criteria.

Tear-depth sensitivity showed that transport limitation became more severe with increasing distance from the red-zone boundary (Figure 4). In the reference condition, the model remained above C/C0 = 0.30 only for shallower tear locations near the vascular side of the domain. By contrast, the augmented sustained-source design example maintained values above C/C0 = 0.30 across the tested depth range, including the 2.5 mm tear. This result is consistent with the known relationship between meniscal vascular zone and repair potential (Arnoczky and Warren, 1982; Mordecai et al., 2014).

**FIGURE 4:**
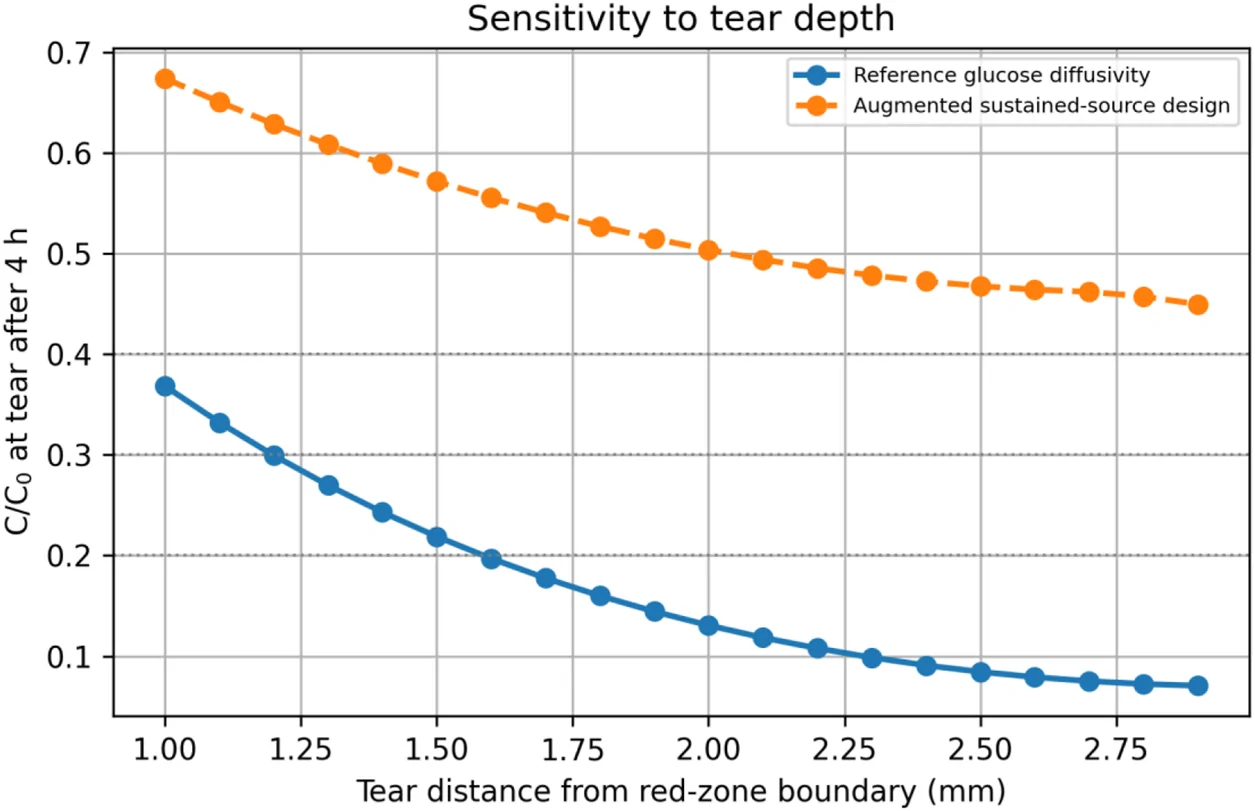
Sensitivity of glucose availability to tear depth. Glucose availability decreases as the tear moves farther from the red-zone boundary. The augmented design example expands the depth range over which the tear environment reaches the fractional comparison levels.

The D-k design-space analysis addressed whether the conclusion depends on one chosen consumption rate (Figure 5). At lower consumption (k/k_ref = 0.5), the model required approximately D/D_ref = 1.59 to reach C/C0 = 0.30. At the reference consumption rate, the requirement increased to approximately D/D_ref = 2.33. When consumption doubled, the required transport enhancement increased to approximately D/D_ref = 4.12. Therefore, the model does not claim that diffusivity alone determines the outcome under all conditions. Instead, it shows that glucose transport estimates depend on the balance between supply through diffusion and loss through consumption.

**FIGURE 5:**
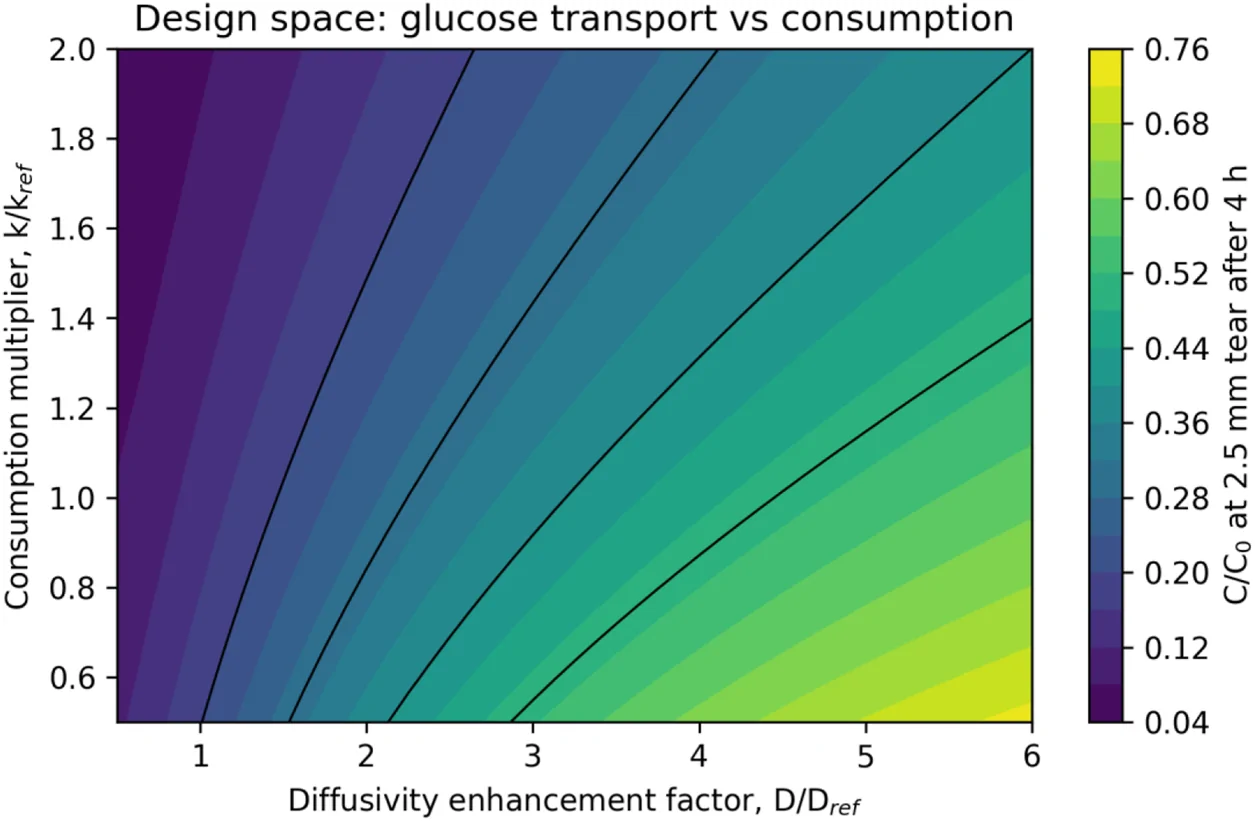
Design-space analysis showing the interaction between glucose diffusivity enhancement and glucose consumption. The contour lines show fractional glucose comparison levels at the tear site after 4 h. When consumption is higher, a larger diffusivity enhancement is required to reach the same comparison level.

Source-release analysis showed that scaffold kinetics can alter predicted tear-site glucose profiles (Figure 6). The sustained-source case reached C/C0 = 0.30 at 97 min and C/C0 = 0.40 at 151 min, with a 4-h tear-site concentration of C/C0 = 0.468. The first-order depletion case reached C/C0 = 0.30 at 101 min and C/C0 = 0.40 at 165 min, with a 4-h concentration of C/C0 = 0.445. The burst-decay case reached C/C0 = 0.30 at 101 min and C/C0 = 0.40 at 162 min, with a 4-h concentration of C/C0 = 0.450. These results show that the constant-source assumption is an optimistic sustained-delivery case, while depletion-based release slightly delays comparison-level crossing and lowers late glucose availability.

**FIGURE 6:**
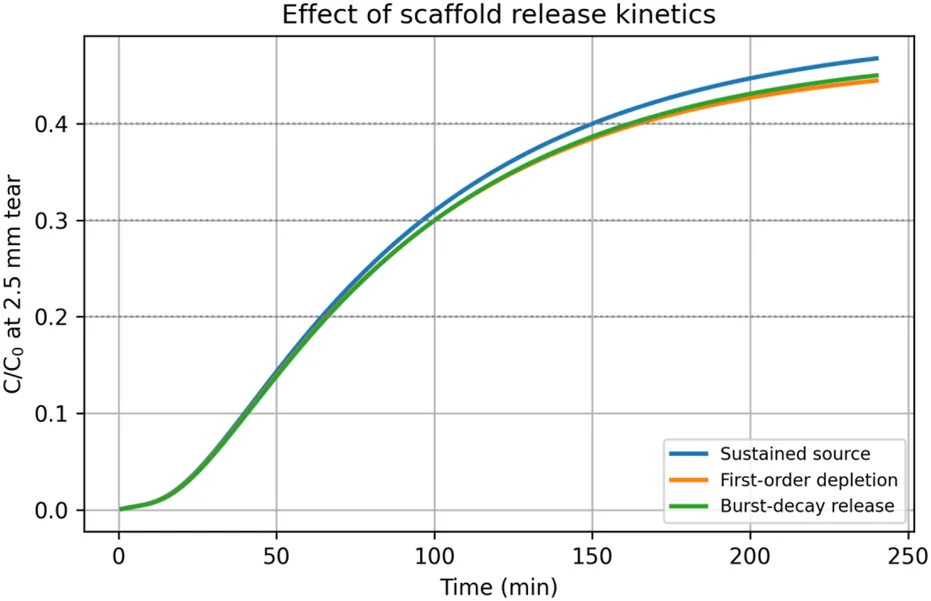
Effect of scaffold release kinetics on tear-site glucose concentration. Constant sustained release gives the highest late concentration, while first-order depletion and burst-decay release delay comparison-level crossing and lower 4-h glucose availability.

Initial-condition sensitivity showed that the main conclusion was not caused only by starting from a nutrient-depleted domain (Figure 7). Under the zero-depleted initial condition, the reference case reached C/C0 = 0.084 after 4 h and the augmented sustained-source case reached C/C0 = 0.468. When the model was initialized with a reference steady glucose profile, the reference case reached C/C0 = 0.127 and the augmented case reached C/C0 = 0.480 after 4 h. The augmented case reached C/C0 = 0.30 earlier under the reference steady initial condition, but the native reference case still remained below C/C0 = 0.30. Therefore, the qualitative conclusion that native reference transport remains limited at a 2.5 mm tear was unchanged.

**FIGURE 7:**
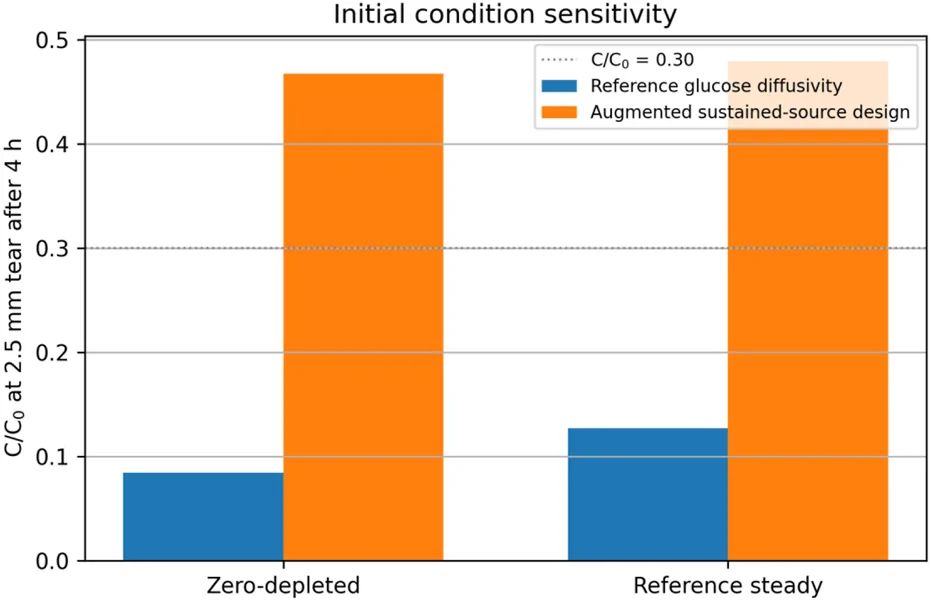
Initial-condition sensitivity. Replacing the zero-depleted initial condition with a reference steady glucose profile increases early tear-site concentration but does not make the native reference case reach C/C0 = 0.30 within 4 h.

Extending the simulation window reduced the concern that the 4-h endpoint alone created the contrast between native and augmented cases (Figure 8). In the reference case, C/C0 at the tear site increased from 0.084 at 4 h to 0.121 at 8 h and 0.127 at 24 h. In the upper glucose diffusivity case, C/C0 increased from 0.163 at 4 h to 0.205 at 8 h and 0.210 at 24 h. Neither native case reached C/C0 = 0.30 even after 24 h. In the augmented sustained-source design example, C/C0 reached 0.468 at 4 h and approached 0.497 by 24 h. The first-order depletion augmented case reached C/C0 = 0.445 at 4 h and approached C/C0 = 0.466 by 24 h. This shows that the native condition was not simply about to cross the comparison level immediately after the simulation stopped.

**FIGURE 8:**
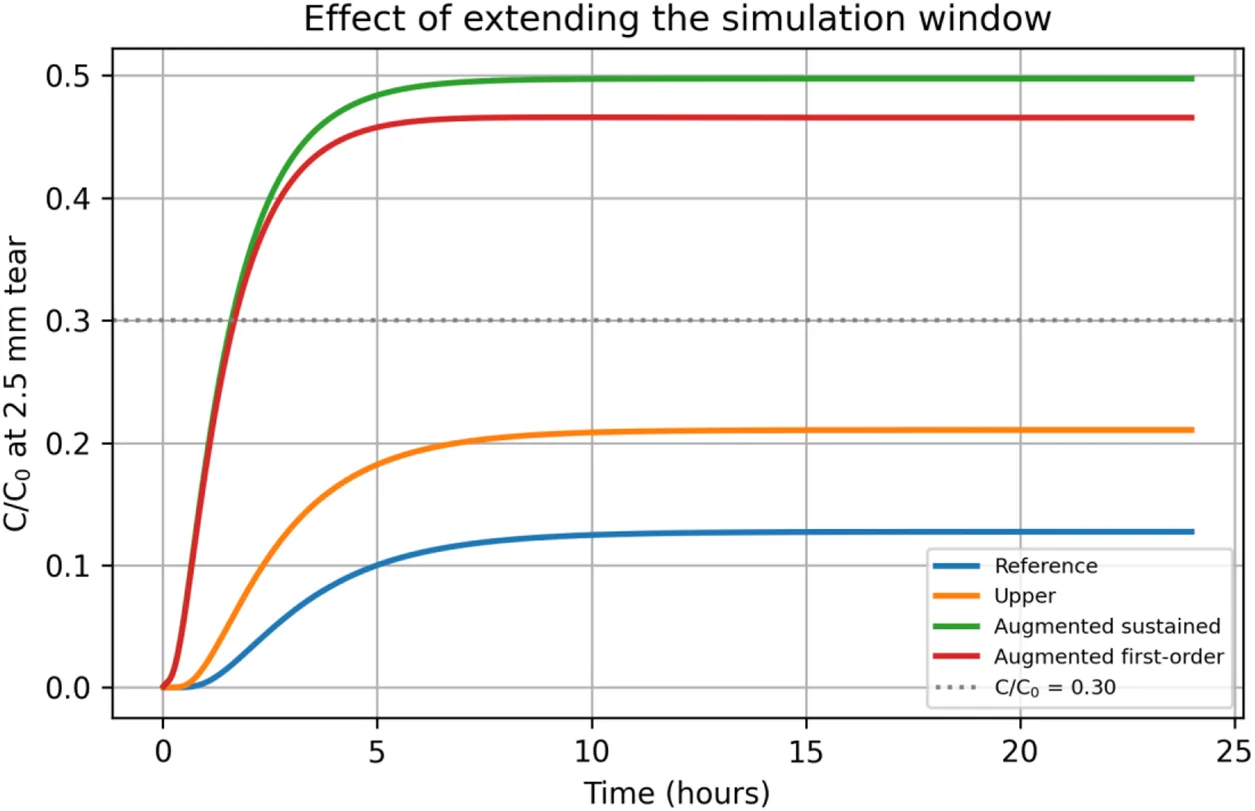
Extended time-window check. The reference and upper native glucose diffusivity cases remain below C/C0 = 0.30 even when simulated to 24 h. The augmented cases cross the comparison level earlier and approach higher late-time values.

Numerical verification showed close agreement between the finite-difference solver and the analytical no-source reaction-diffusion solution (Figure 9). The relative L2 error between the numerical 48-h profile and the analytical steady-state profile was 1.22 × 10^−5^. This supports correct implementation of the diffusion-consumption equation under simplified conditions, although it does not biologically validate the model predictions.

**FIGURE 9:**
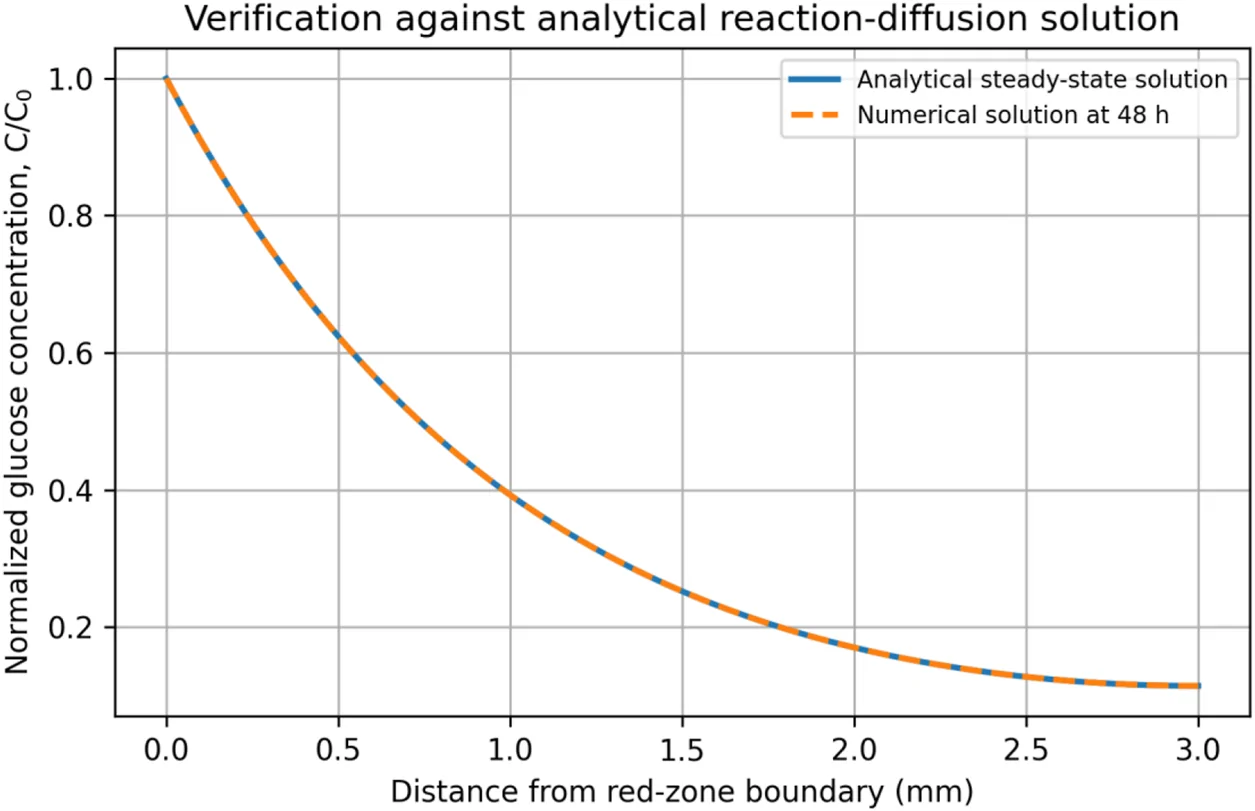
Verification against an analytical reaction-diffusion solution. The numerical solution closely matches the analytical steady-state solution for the no-source case with fixed red-zone boundary and zero-flux inner boundary.

Numerical convergence analysis showed that the late-time glucose profiles were changing slowly by the end of the 4-h simulations (Figure 10). The final relative profile change was below 10^−3^ for both the reference and augmented simulations. Because exact biological steady state was not the focus, outputs were described as late-time model predictions rather than definitive physiological equilibria.

**FIGURE 10:**
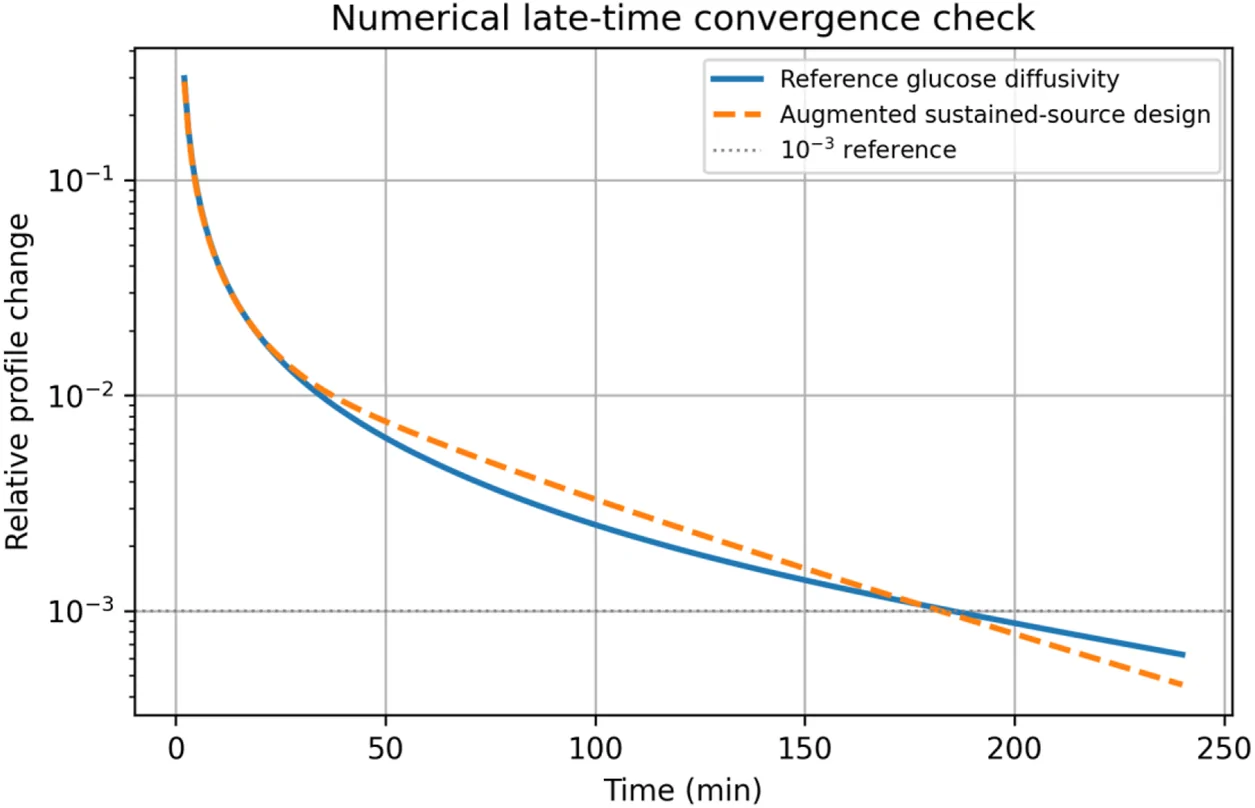
Numerical late-time convergence check. Relative profile change decreased over time for both reference and augmented simulations. Final outputs are interpreted as stable late-time numerical profiles, not as proof of biological steady state.

## Discussion

This study developed a glucose-specific computational framework to examine whether transport enhancement could improve the glucose environment of a deep white-zone meniscal tear. The central scientific value is not the expected observation that higher diffusivity increases diffusion. The useful output is the set of model-derived estimates: how much diffusivity enhancement is needed at a specific tear depth, how that estimate changes with local supplementation, how mathematical comparison levels affect interpretation, how release kinetics alter glucose profiles, how initial conditions influence early results, and how consumption changes the required transport enhancement.

Specifying glucose directly addresses the limitation of using a generic nutrient variable. Oxygen, glucose, glutamine, cytokines, and growth factors differ in molecular size, diffusivity, solubility, reaction kinetics, and cellular consumption. A single generic nutrient cannot be assigned one physiologically meaningful diffusivity and one consumption rate. For this reason, C(x,t) is now interpreted as normalized glucose concentration. The diffusivity values in Table 2 are taken from glucose diffusion measurements in meniscus fibrocartilage, and any future oxygen or glutamine version of the model would require molecule-specific D and k values (Kleinhans et al., 2015; Travascio and Jackson, 2017).

The fractional comparison levels are central to the revised interpretation. Since an exact glucose concentration that guarantees meniscal healing has not been established, C/C0 = 0.30 should not be interpreted as a biological healing threshold. It is better understood as an intermediate mathematical comparison level. The broader range from C/C0 = 0.20 to 0.50 gives a more honest picture of the model output. Because the values are normalized to red-zone glucose availability, they connect the model output to fractional glucose restoration, not to a validated clinical endpoint. This approach also makes the model easier to validate experimentally because future studies can replace the normalized comparison levels with measured glucose concentrations linked to cell viability, matrix synthesis, inflammatory signaling, or other biological readouts (Adesida et al., 2006; Adesida et al., 2007; Hennerbichler et al., 2007; Szojka et al., 2019).

The boundary conditions require careful interpretation. The fixed red-zone boundary represents a vascular-side glucose supply, while the zero-flux inner boundary represents the end of the simplified radial domain. Synovial fluid was not included as a separate supply source because the model is not a full three-dimensional meniscus geometry and does not include the superior and inferior articular surfaces that contact synovial fluid. Travascio and Jackson (2017) showed that glucose supply to the meniscus can involve both synovial fluid and peripheral vascular supply, so omitting synovial surface exchange is a limitation. The present framework therefore should be interpreted as a conservative radial transport model, not as a complete model of all *in vivo* glucose sources.

The initial-condition analysis also improves interpretation. The zero-depleted initial condition is useful for comparing early transport rescue, but it is not a physiological resting state. When a reference steady glucose profile was used as the starting condition, the native reference case still remained below C/C0 = 0.30 at 4 h, while the augmented case remained higher. This suggests that the main result was not simply an artifact of initializing the tissue interior at zero concentration.

The D-k design-space result addresses the limitation of using a constant glucose consumption rate. A single first-order k cannot represent all regional metabolic states in injured meniscus. Cell density, cell phenotype, tissue region, inflammation, macrophage infiltration, cytokines such as IL-1 and TNF-alpha, and matrix damage may all alter glucose uptake and metabolic demand. Inflammatory cytokines have been shown to inhibit meniscal repair *in vitro*, and cytokine signaling can affect glucose transport and metabolism in chondrocyte-like cells and joint tissues (Hennerbichler et al., 2007; Shikhman et al., 2001; Yan et al., 2021). The current model therefore uses k_ref only as a reference consumption term and varies k/k_ref to show how transport estimates change when consumption is lower or higher. Future models should replace constant k with k(x,t) or k(C, inflammation, cell density) once experimental data are available.

The source-kinetics analysis addresses another important limitation. A constant source is not a realistic description of many polymer matrices or hydrogels. Scaffold-based systems frequently show an early burst, diffusion-controlled release, swelling-controlled release, or degradation-dependent depletion (Higuchi, 1961; Huang and Brazel, 2001; Siepmann and Siepmann, 2012; Whitaker et al., 2001). In the present model, time-dependent source depletion delayed comparison-level crossing and lowered late concentration compared with sustained release. This means constant S0 should be interpreted as an idealized sustained-delivery upper bound, not as a prediction for all biomaterial systems.

The model-derived diffusivity estimates should not be interpreted as clinically applicable scaffold criteria. The estimated D/D_ref values depend on the selected glucose diffusivity, consumption rate, initial condition, source term, source kinetics, tear depth, boundary conditions, and simplified one-dimensional geometry. The values are useful because they define experimentally testable hypotheses. A future scaffold experiment could measure whether a porous scaffold, microchannel pattern, trephination-like pathway, or hydrogel delivery system changes effective glucose transport by a comparable fold-change, but the present model alone cannot establish a clinical scaffold requirement.

The previous vascular-response variable was removed from the model because the current equation did not include the anti-angiogenic environment of the white zone and could be misread as a prediction of vascularization. The inner meniscus contains anti-angiogenic mechanisms that help preserve avascularity, including endostatin and chondromodulin-I-related effects (Fujii et al., 2013; Petersen et al., 2005; Yan et al., 2021). A valid vascular model would need to include pro-angiogenic and anti-angiogenic signaling, endothelial cell migration, matrix inhibition, vessel remodeling, and longer biological time scales. The present framework therefore focuses only on glucose transport, while vascular remodeling is treated as outside the scope of this study.

Several structural limitations remain. The model is one-dimensional and treats the meniscus as a homogeneous radial slab, even though the real tissue is a three-dimensional wedge-shaped fibrocartilage with circumferential collagen alignment, radial tie fibers, superficial and deep layers, anisotropic permeability, regional composition differences, and complex interstitial fluid flow during mechanical loading (Fox et al., 2012; Makris et al., 2011; Mameri et al., 2022; Morejon et al., 2021; Schwartz et al., 2022). A one-dimensional model is useful for isolating radial glucose transport to a white-zone tear, but it cannot represent full three-dimensional meniscal geometry, load-induced convection, synovial surface exchange, anisotropic transport tensors, or spatially varying scaffold-tissue integration.

The most direct next step is *ex vivo* validation. Human or sheep meniscus explants could be prepared with a standardized tear or channel at a known distance from the red-zone side. Native tissue, trephination-like channels, porous scaffold conditions, and hydrogel delivery conditions could be compared using glucose assays, fluorescent tracer diffusion, oxygen measurements, or released-solute concentration. The measured effective diffusivity and release functions could then be substituted directly into this model. Cell viability, matrix synthesis, inflammatory markers, and histologic vascular markers could be used to replace normalized mathematical comparison levels with biological endpoints.

## Conclusion

Glucose transport may be an important design constraint for deep white-zone meniscal repair because the modeled tear region remained transport-limited under literature-informed native diffusivity cases. Across the comparison levels tested, the native glucose cases stayed below C/C0 = 0.30 at a 2.5 mm tear, even after the extended 24-h simulation and the reference steady-initial-condition test. The augmented sustained-source example produced higher tear-site glucose availability, but the model-derived enhancement estimates depended on source strength, release kinetics, tear depth, boundary assumptions, initial concentration, and glucose consumption. These outputs should be interpreted as computational estimates, not validated healing thresholds, clinical scaffold requirements, or proof of biological repair. Overall, the framework provides a focused way to compare glucose-transport assumptions, guide *ex vivo* scaffold and explant experiments, and identify which transport measurements should be validated before making biological or clinical claims.

## Impact statement

This work presents a computational framework for evaluating glucose transport as a design constraint in white zone meniscal tissue. Rather than treating a concentration value as a definitive healing threshold, the model reports fractional glucose-restoration comparison levels and estimates how diffusivity, local supplementation, release behavior, tear depth, initial conditions, and glucose consumption shape tear-site concentration. The framework is intended to guide future *ex vivo* diffusion and scaffold testing, not to provide direct clinical thresholds.

## Data Availability

The original contributions presented in the study are included in the article/Supplementary Material, further inquiries can be directed to the corresponding author.
