# Supplementary material for: Glucose transport as a design constraint in white-zone meniscus tissue engineering: a reaction-diffusion framework
Source: Front Bioeng Biotechnol. 2026 Oct 15;14:1880978. doi: 10.3389/fbioe.2026.1880978 (PMC13716718; doi:10.3389/fbioe.2026.1880978)
Supplement: Supplementary file 1 [file DataSheet1.pdf]

```
%% MENISCUS GLUCOSE TRANSPORT MODEL
clear; clc; close all;
%% ------------------------ MODEL PARAMETERS ------------------------
L = 3e-3;                       % radial meniscus domain [m]
N = 150;                        % spatial intervals
x = linspace(0,L,N+1)';
dx = L/N;
C0 = 1.0;                       % normalized red-zone glucose boundary
% Glucose diffusivity values [m^2/s]. These are based on the glucose
% diffusion measurements reported by Kleinhans et al. (2015) for porcine
% meniscus fibrocartilage under different compressive strain conditions.
D_low = 0.79e-10;               % axial-central glucose diffusivity at 20%
compression
D_ref = 1.10e-10;               % axial-central glucose diffusivity at 10%
compression
D_upper = 1.68e-10;             % axial-central glucose diffusivity at 0%
compression
D_aug = 4*D_ref;                % model-derived augmented transport example
k_ref = 1e-4;                   % reference first-order glucose consumption
[s^-1]
x_tear = 2.5e-3;                % deep white-zone tear location [m]
source_width = 0.5e-3;          % local source width [m]
S_aug = 2e-5;                   % example local glucose-equivalent
supplementation [C0/s]
T_end = 4*3600;                 % 4-hour early transport window [s]
dt = 60;                        % implicit time step [s]
benchmarks = [0.20 0.30 0.40 0.50];
idx_tear = nearest_index(x,x_tear);
outdir = 'meniscus_editor_revision_figures';
if ~exist(outdir,'dir')
   mkdir(outdir);
end
%% ------------------------ PARAMETER SCALING CHECKS ------------------------
% These checks quantify the time scale, length scale, and source strength
% implied by the selected phenomenological values. They do not biologically
% validate the parameters.
T4 = T_end;
k_time_h = (1/k_ref)/3600;
k_half_h = log(2)/k_ref/3600;
ell_ref_mm = sqrt(D_ref/k_ref)*1e3;
Da_tear = k_ref*x_tear^2/D_ref;
source_equilibrium = S_aug/k_ref;
source_only_4h = source_equilibrium*(1 - exp(-k_ref*T4));
fprintf('\nQuantitative parameter-scaling checks:\n');
fprintf('k_ref: 1/k = %.2f h, half-life = %.2f h, sqrt(D_ref/k_ref) = %.2f mm,
k*x_tear^2/D_ref = %.2f\n', ...
   k_time_h,k_half_h,ell_ref_mm,Da_tear);
fprintf('S0: S0/k_ref = %.3f C0, source-only C(4 h) = %.3f
C0\n',source_equilibrium,source_only_4h);
```

```
%% ------------------------ MAIN SIMULATIONS ------------------------
cases(1).name = 'Lower glucose diffusivity';        cases(1).D = D_low;
cases(1).S0 = 0;     cases(1).source_mode = 'none';
cases(2).name = 'Reference glucose diffusivity';    cases(2).D = D_ref;
cases(2).S0 = 0;     cases(2).source_mode = 'none';
cases(3).name = 'Upper glucose diffusivity';        cases(3).D = D_upper;
cases(3).S0 = 0;     cases(3).source_mode = 'none';
cases(4).name = 'Augmented sustained-source design';cases(4).D = D_aug;
cases(4).S0 = S_aug; cases(4).source_mode = 'constant';
for c = 1:numel(cases)
   cases(c).out = run_model(cases(c).D,cases(c).S0,L,N,C0,k_ref, ...
       x_tear,source_width,T_end,dt,cases(c).source_mode,'zero',true);
end
fprintf('\nFinal tear-site glucose values at x = %.1f mm after 4
h:\n',x_tear*1e3);
for c = 1:numel(cases)
   fprintf('%s: C/C0 = %.3f\n',cases(c).name,cases(c).out.C(idx_tear));
end
%% ------------------------ FIGURE 1: GLUCOSE PROFILES
-------------------------
figure('Color','w'); hold on;
for c = 1:numel(cases)
   plot(x*1e3,cases(c).out.C,'LineWidth',2);
end
yline(0.30,':','C/C0 = 0.30 comparison level','LineWidth',1.2);
xline(x_tear*1e3,'-.','Tear site','LineWidth',1.0);
xlabel('Distance from red-zone boundary (mm)');
ylabel('Normalized glucose concentration, C/C_0');
title('Glucose profiles at 4 h');
legend({cases.name,'C/C0 = 0.30 comparison level','Tear
site'},'Location','best','FontSize',8);
grid on; box on;
save_figure(gcf,fullfile(outdir,'figure1_glucose_profiles.png'));
%% ------------------------ FIGURE 2: COMPARISON LEVEL TIME COURSE
-------------------------
ref = cases(2).out;
aug = cases(4).out;
figure('Color','w'); hold on;
plot(ref.t/60,ref.Ctear,'LineWidth',2);
plot(aug.t/60,aug.Ctear,'--','LineWidth',2);
for b = benchmarks
   yline(b,':',sprintf('C/C0 = %.1f',b),'LineWidth',1.0);
end
xlabel('Time (min)');
ylabel('C/C_0 at 2.5 mm tear');
title('Tear-site glucose time course and comparison levels');
legend('Reference glucose diffusivity','Augmented sustained-source
design','Location','best','FontSize',8);
grid on; box on;
```

```
save_figure(gcf,fullfile(outdir,'figure2_benchmark_timecourse.png'));
fprintf('\nCrossing times for augmented sustained-source design:\n');
for b = benchmarks
   t_cross = time_to_benchmark(aug.t,aug.Ctear,b);
   if isnan(t_cross)
       fprintf('C/C0 = %.2f: not reached within 4 h\n',b);
   else
       fprintf('C/C0 = %.2f: %.1f min\n',b,t_cross/60);
   end
end
%% ------------------------ FIGURE 3: MODEL-DERIVED DIFFUSIVITY ESTIMATE
-------------------------
Svals = [0 0.5e-5 1e-5 2e-5 3e-5];
Dfolds = linspace(0.25,12,80);
D_est = nan(numel(benchmarks),numel(Svals));
for s = 1:numel(Svals)
   for b = 1:numel(benchmarks)
       for f = 1:numel(Dfolds)
           out = run_model(Dfolds(f)*D_ref,Svals(s),L,N,C0,k_ref, ...
               x_tear,source_width,T_end,dt,'constant','zero',false);
           if out.C(idx_tear) >= benchmarks(b)
               D_est(b,s) = Dfolds(f);
               break;
           end
       end
   end
end
figure('Color','w'); hold on;
for b = 1:numel(benchmarks)
   plot(Svals/1e-5,D_est(b,:),'o-','LineWidth',2);
end
xlabel('Local source strength S_0 (×10^{-5} C_0/s)');
ylabel('Model-derived diffusivity enhancement, D/D_{ref}');
title('Model-derived diffusivity estimate at a 2.5 mm tear');
legend('C/C0 = 0.2','C/C0 = 0.3','C/C0 = 0.4','C/C0 = 0.5','Location','best');
grid on; box on;
save_figure(gcf,fullfile(outdir,'figure3_diffusivity_estimate.png'));
%% ------------------------ FIGURE 4: TEAR DEPTH SENSITIVITY
-------------------------
depths = linspace(1.0e-3,2.9e-3,20);
Cdepth_ref = zeros(size(depths));
Cdepth_aug = zeros(size(depths));
for i = 1:numel(depths)
   temp =
run_model(D_ref,0,L,N,C0,k_ref,depths(i),source_width,T_end,dt,'none','zero',f
alse);
   Cdepth_ref(i) = temp.C(nearest_index(x,depths(i)));
```

```
   temp =
run_model(D_aug,S_aug,L,N,C0,k_ref,depths(i),source_width,T_end,dt,'constant',
'zero',false);
   Cdepth_aug(i) = temp.C(nearest_index(x,depths(i)));
end
figure('Color','w'); hold on;
plot(depths*1e3,Cdepth_ref,'o-','LineWidth',2);
plot(depths*1e3,Cdepth_aug,'o--','LineWidth',2);
yline(0.20,':','C/C0 = 0.2','LineWidth',1.0);
yline(0.30,':','C/C0 = 0.3','LineWidth',1.0);
yline(0.40,':','C/C0 = 0.4','LineWidth',1.0);
xlabel('Tear distance from red-zone boundary (mm)');
ylabel('C/C_0 at tear after 4 h');
title('Sensitivity to tear depth');
legend('Reference glucose diffusivity','Augmented sustained-source
design','Location','best','FontSize',8);
grid on; box on;
save_figure(gcf,fullfile(outdir,'figure4_tear_depth_sensitivity.png'));
%% ------------------------ FIGURE 5: DIFFUSIVITY-CONSUMPTION DESIGN SPACE
-------------------------
D_folds_grid = linspace(0.5,6,45);
k_mults = linspace(0.5,2.0,35);
Z = zeros(numel(k_mults),numel(D_folds_grid));
for i = 1:numel(k_mults)
   for j = 1:numel(D_folds_grid)
       out = run_model(D_folds_grid(j)*D_ref,S_aug,L,N,C0,k_ref*k_mults(i),
...
           x_tear,source_width,T_end,dt,'constant','zero',false);
       Z(i,j) = out.C(idx_tear);
   end
end
figure('Color','w');
contourf(D_folds_grid,k_mults,Z,20,'LineStyle','none'); hold on;
colorbar;
contour(D_folds_grid,k_mults,Z,[0.20 0.30 0.40 0.50],'k','LineWidth',0.8);
xlabel('Diffusivity enhancement factor, D/D_{ref}');
ylabel('Consumption multiplier, k/k_{ref}');
title('Design space: glucose transport vs consumption');
grid on; box on;
save_figure(gcf,fullfile(outdir,'figure5_design_space_D_k.png'));
%% ------------------------ FIGURE 6: SCAFFOLD RELEASE KINETICS
-------------------------
release_cases(1).name = 'Sustained source';        release_cases(1).mode =
'constant';
release_cases(2).name = 'First-order depletion';  release_cases(2).mode =
'first_order';
release_cases(3).name = 'Burst-decay release';    release_cases(3).mode =
'burst_decay';
figure('Color','w'); hold on;
```

```
for r = 1:numel(release_cases)
   release_cases(r).out = run_model(D_aug,S_aug,L,N,C0,k_ref, ...
       x_tear,source_width,T_end,dt,release_cases(r).mode,'zero',false);
   plot(release_cases(r).out.t/60,release_cases(r).out.Ctear,'LineWidth',2);
end
for b = [0.20 0.30 0.40]
   yline(b,':',sprintf('C/C0 = %.1f',b),'LineWidth',1.0);
end
xlabel('Time (min)');
ylabel('C/C_0 at 2.5 mm tear');
title('Effect of scaffold release kinetics');
legend({release_cases.name},'Location','best','FontSize',8);
grid on; box on;
save_figure(gcf,fullfile(outdir,'figure6_release_kinetics.png'));
%% ------------------------ FIGURE 7: INITIAL CONDITION SENSITIVITY
-------------------------
init_cases(1).name = 'Zero-depleted initial condition'; init_cases(1).mode =
'zero';
init_cases(2).name = 'Reference steady initial condition'; init_cases(2).mode
= 'steady_ref';
Cinit_ref = zeros(1,2);
Cinit_aug = zeros(1,2);
T03_aug = zeros(1,2);
for i = 1:2
   temp =
run_model(D_ref,0,L,N,C0,k_ref,x_tear,source_width,T_end,dt,'none',init_cases(
i).mode,false);
   Cinit_ref(i) = temp.C(idx_tear);
   temp =
run_model(D_aug,S_aug,L,N,C0,k_ref,x_tear,source_width,T_end,dt,'constant',ini
t_cases(i).mode,false);
   Cinit_aug(i) = temp.C(idx_tear);
   T03_aug(i) = time_to_benchmark(temp.t,temp.Ctear,0.30)/60;
end
figure('Color','w');
b = bar([Cinit_ref; Cinit_aug]');
yline(0.30,':','C/C0 = 0.30','LineWidth',1.0);
set(gca,'XTickLabel',{'Zero-depleted','Reference steady'});
ylabel('C/C_0 at 2.5 mm tear after 4 h');
title('Initial condition sensitivity');
legend('Reference glucose diffusivity','Augmented sustained-source
design','Location','best','FontSize',8);
grid on; box on;
save_figure(gcf,fullfile(outdir,'figure7_initial_condition_sensitivity.png'));
%% ------------------------ FIGURE 8: EXTENDED TIME WINDOW CHECK
-------------------------
window_cases(1).name = 'Reference glucose diffusivity';   window_cases(1).D =
D_ref;   window_cases(1).S0 = 0;     window_cases(1).mode = 'none';
```

```
window_cases(2).name = 'Upper glucose diffusivity';       window_cases(2).D =
D_upper; window_cases(2).S0 = 0;     window_cases(2).mode = 'none';
window_cases(3).name = 'Augmented sustained source';      window_cases(3).D =
D_aug;   window_cases(3).S0 = S_aug; window_cases(3).mode = 'constant';
window_cases(4).name = 'Augmented first-order depletion'; window_cases(4).D =
D_aug;   window_cases(4).S0 = S_aug; window_cases(4).mode = 'first_order';
figure('Color','w'); hold on;
for c = 1:numel(window_cases)
   window_cases(c).out =
run_model(window_cases(c).D,window_cases(c).S0,L,N,C0,k_ref, ...
       x_tear,source_width,24*3600,dt,window_cases(c).mode,'zero',false);
   plot(window_cases(c).out.t/3600,window_cases(c).out.Ctear,'LineWidth',2);
end
yline(0.30,':','C/C0 = 0.30','LineWidth',1.2);
xlabel('Time (hours)');
ylabel('C/C_0 at 2.5 mm tear');
title('Effect of extending the simulation window');
legend({window_cases.name},'Location','best','FontSize',7);
grid on; box on;
save_figure(gcf,fullfile(outdir,'figure8_time_window_check.png'));
%% ------------------------ FIGURE 9: ANALYTICAL VERIFICATION
------------------------
verify_out =
run_model(D_ref,0,L,N,C0,k_ref,x_tear,source_width,48*3600,dt,'none','zero',fa
lse);
lambda = sqrt(k_ref/D_ref);
C_analytic = C0*cosh(lambda*(L-x))/cosh(lambda*L);
rel_error = norm(verify_out.C - C_analytic)/norm(C_analytic);
figure('Color','w'); hold on;
plot(x*1e3,C_analytic,'LineWidth',2);
plot(x*1e3,verify_out.C,'--','LineWidth',2);
xlabel('Distance from red-zone boundary (mm)');
ylabel('Normalized glucose concentration, C/C_0');
title('Verification against analytical reaction-diffusion solution');
legend('Analytical steady-state solution','Numerical solution at 48
h','Location','best','FontSize',8);
grid on; box on;
save_figure(gcf,fullfile(outdir,'figure9_analytical_verification.png'));
fprintf('\nAnalytical verification relative L2 error = %.3e\n',rel_error);
%% ------------------------ FIGURE 10: NUMERICAL CONVERGENCE
------------------------
figure('Color','w'); hold on;
semilogy(ref.eps_t/60,ref.eps,'LineWidth',2);
semilogy(aug.eps_t/60,aug.eps,'--','LineWidth',2);
yline(1e-3,':','10^{-3} reference','LineWidth',1.2);
xlabel('Time (min)');
ylabel('Relative profile change');
title('Numerical late-time convergence check');
```

```
legend('Reference glucose diffusivity','Augmented sustained-source
design','Location','best');
grid on; box on;
save_figure(gcf,fullfile(outdir,'figure10_convergence.png'));
%% ------------------------ PRINT SUMMARY -------------------------
fprintf('\nModel-derived diffusivity enhancement D/Dref needed at x = %.1f mm
after 4 h:\n',x_tear*1e3);
fprintf('Rows = comparison levels %.2f %.2f %.2f %.2f; columns = S0 ×10^-5 =
0, 0.5, 1, 2, 3\n',benchmarks);
disp(D_est);
fprintf('\nInitial condition sensitivity summary:\n');
for i = 1:2
   fprintf('%s: reference C4h = %.3f, augmented C4h = %.3f, augmented t0.30 =
%.1f min\n', ...
       init_cases(i).name,Cinit_ref(i),Cinit_aug(i),T03_aug(i));
end
%% ------------------------ LOCAL FUNCTIONS -------------------------
function out =
run_model(D,S0,L,N,C0,k,x_tear,source_width,T_end,dt,source_mode,initial_mode,
do_convergence)
   dx = L/N;
   x = linspace(0,L,N+1)';
   xi = x(2:end);
   idx_tear = nearest_index(x,x_tear);
   idx_unknown = max(1,idx_tear-1);
   e = ones(N,1);
   Lmat = spdiags([e -2*e e],[-1 0 1],N,N)/(dx^2);
   Lmat(end,end-1) = 2/(dx^2);  % zero-flux radial inner boundary
   I = speye(N);
   bcC = zeros(N,1);
   bcC(1) = C0/(dx^2);
   source_mask = double(abs(xi - x_tear) <= source_width/2);
   A_C = I - dt*(D*Lmat - k*I);
   if strcmp(initial_mode,'zero')
       C = zeros(N,1);
   elseif strcmp(initial_mode,'steady_ref')
       D_ref_local = 1.10e-10;
       lambda = sqrt(k/D_ref_local);
       Cfull = C0*cosh(lambda*(L-x))/cosh(lambda*L);
       C = Cfull(2:end);
   else
       error('Unknown initial_mode. Use zero or steady_ref.');
   end
   Nt = round(T_end/dt);
   t = zeros(Nt,1);
   Ctear = zeros(Nt,1);
   conv_err = [];
   conv_t = [];
   Cprev = [];
```

```
    small_number = 1e-12;
    for n = 1:Nt
        current_time = n*dt;
        S = source_mask * source_amplitude(current_time,S0,source_mode);
        C = A_C \ (C + dt*(D*bcC + S));
        t(n) = current_time;
        Ctear(n) = C(idx_unknown);
        if do_convergence
            if ~isempty(Cprev)
                conv_err(end+1,1) = norm(C-Cprev)/(norm(C)+small_number);
%#ok<AGROW>
                conv_t(end+1,1) = current_time; %#ok<AGROW>
            end
            Cprev = C;
        end
    end
    out.C = [C0; C];
    out.t = t;
    out.Ctear = Ctear;
    out.eps = conv_err;
    out.eps_t = conv_t;
end
function S_t = source_amplitude(t,S0,source_mode)
    switch source_mode
        case 'none'
            S_t = 0;
        case 'constant'
            S_t = S0;
        case 'first_order'
            tau_release = 2*3600;
            S_t = S0*exp(-t/tau_release);
        case 'burst_decay'
            burst_fraction = 0.40;
            tau_fast = 0.5*3600;
            tau_slow = 8*3600;
            S_t = S0*(burst_fraction*exp(-t/tau_fast) +
(1-burst_fraction)*exp(-t/tau_slow));
        otherwise
            error('Unknown source_mode. Use none, constant, first_order, or
burst_decay.');
    end
end
function idx = nearest_index(x,value)
    [~,idx] = min(abs(x-value));
end
function t_cross = time_to_benchmark(t,Ctear,benchmark)
    idx = find(Ctear >= benchmark,1,'first');
    if isempty(idx)
        t_cross = NaN;
```

```
    else
        t_cross = t(idx);
    end
end
function save_figure(fig_handle,filename)
    try
        exportgraphics(fig_handle,filename,'Resolution',300);
    catch
        print(fig_handle,filename,'-dpng','-r300');
    end
end
```
